# Supplementary material for: A Humanized Yeast Phenomic Model of Deoxycytidine Kinase to Predict Genetic Buffering of Nucleoside Analog Cytotoxicity
Source: Genes (Basel). 2019 Sep 30;10(10):770. doi: 10.3390/genes10100770 (PMC6826991; doi:10.3390/genes10100770)
Supplement: Supplementary file 1 [file genes-10-00770-s001.zip › Additional_Files/Additional_File7_GOTermHeatmaps.zip!/Additional_File7_GOTermHeatmaps/vesicle_fusion.pdf]

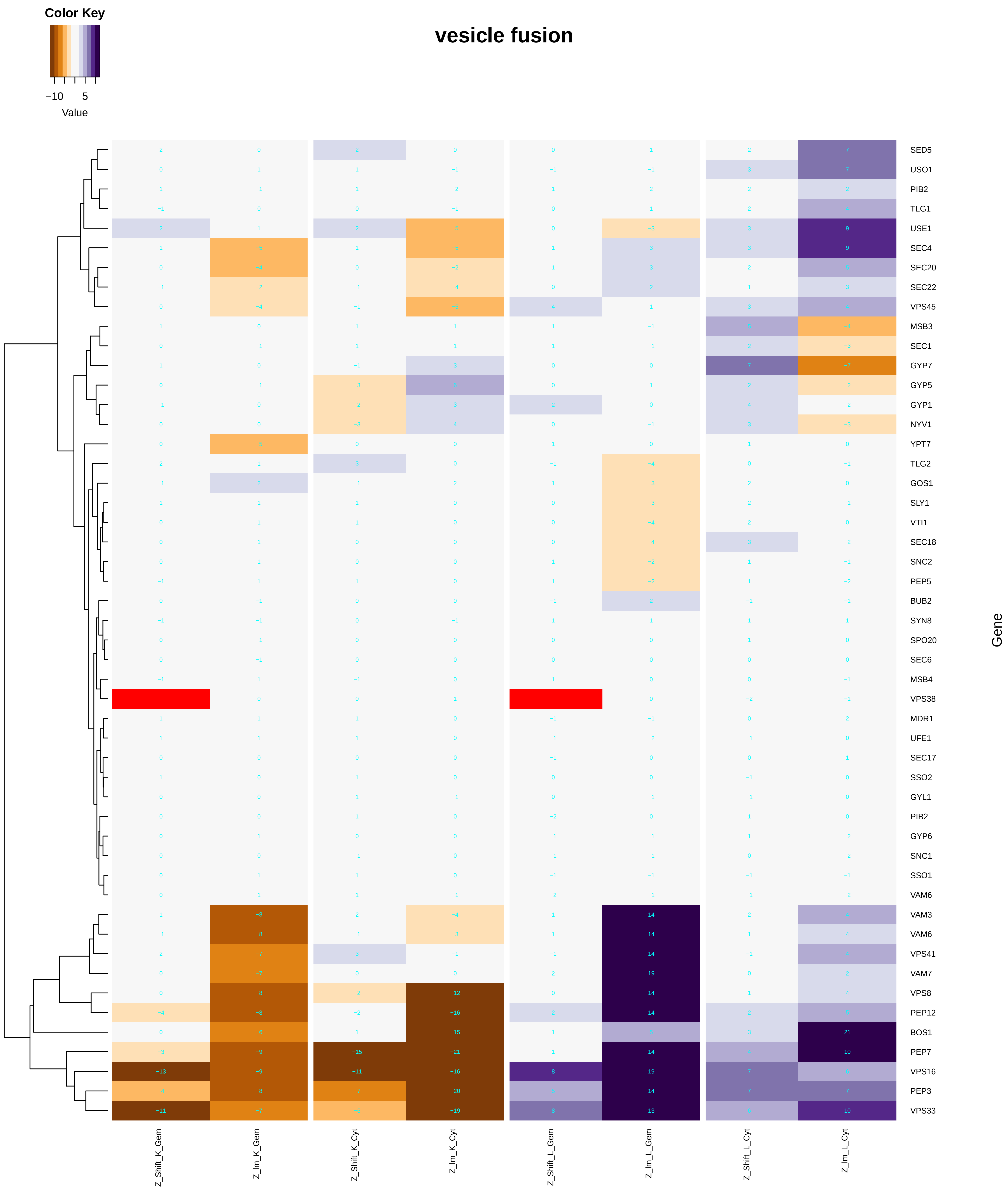
vesicle fusion
Color Key
−10
5
Value
Gene
SED5
USO1
PIB2
TLG1
USE1
SEC4
SEC20
SEC22
VPS45
MSB3
SEC1
GYP7
GYP5
GYP1
NYV1
YPT7
TLG2
GOS1
SLY1
VTI1
SEC18
SNC2
PEP5
BUB2
SYN8
SPO20
SEC6
MSB4
VPS38
MDR1
UFE1
SEC17
SSO2
GYL1
PIB2
GYP6
SNC1
SSO1
VAM6
VAM3
VAM6
VPS41
VAM7
VPS8
PEP12
BOS1
PEP7
VPS16
PEP3
VPS33
Z_Shift_K_Gem
Z_Im_K_Gem
Z_Shift_K_Cyt
Z_Im_K_Cyt
Z_Shift_L_Gem
Z_Im_L_Gem
Z_Shift_L_Cyt
Z_Im_L_Cyt

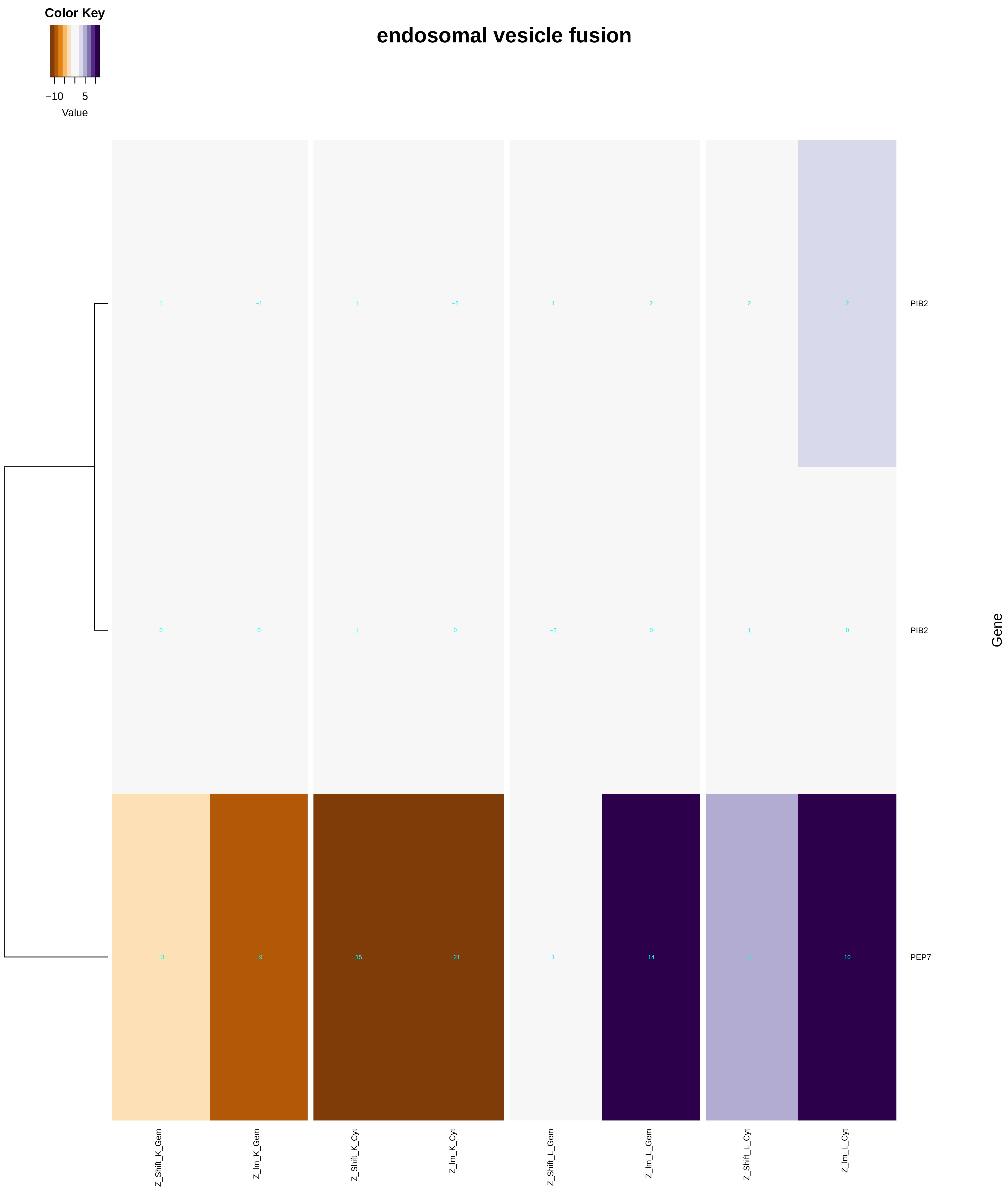
Color Key
−10
5
Value
endosomal vesicle fusion
1
−1
1
−2
1
2
2
2
PIB2
0
0
1
0
−2
0
1
0
PIB2
−3
−9
−15
−21
1
14
10
PEP7
Gene
Z_Shift_K_Gem
Z_Im_K_Gem
Z_Shift_K_Cyt
Z_Im_K_Cyt
Z_Shift_L_Gem
Z_Im_L_Gem
Z_Shift_L_Cyt
Z_Im_L_Cyt

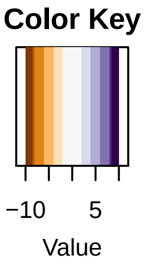

vesicle fusion with endoplasmic reticulum

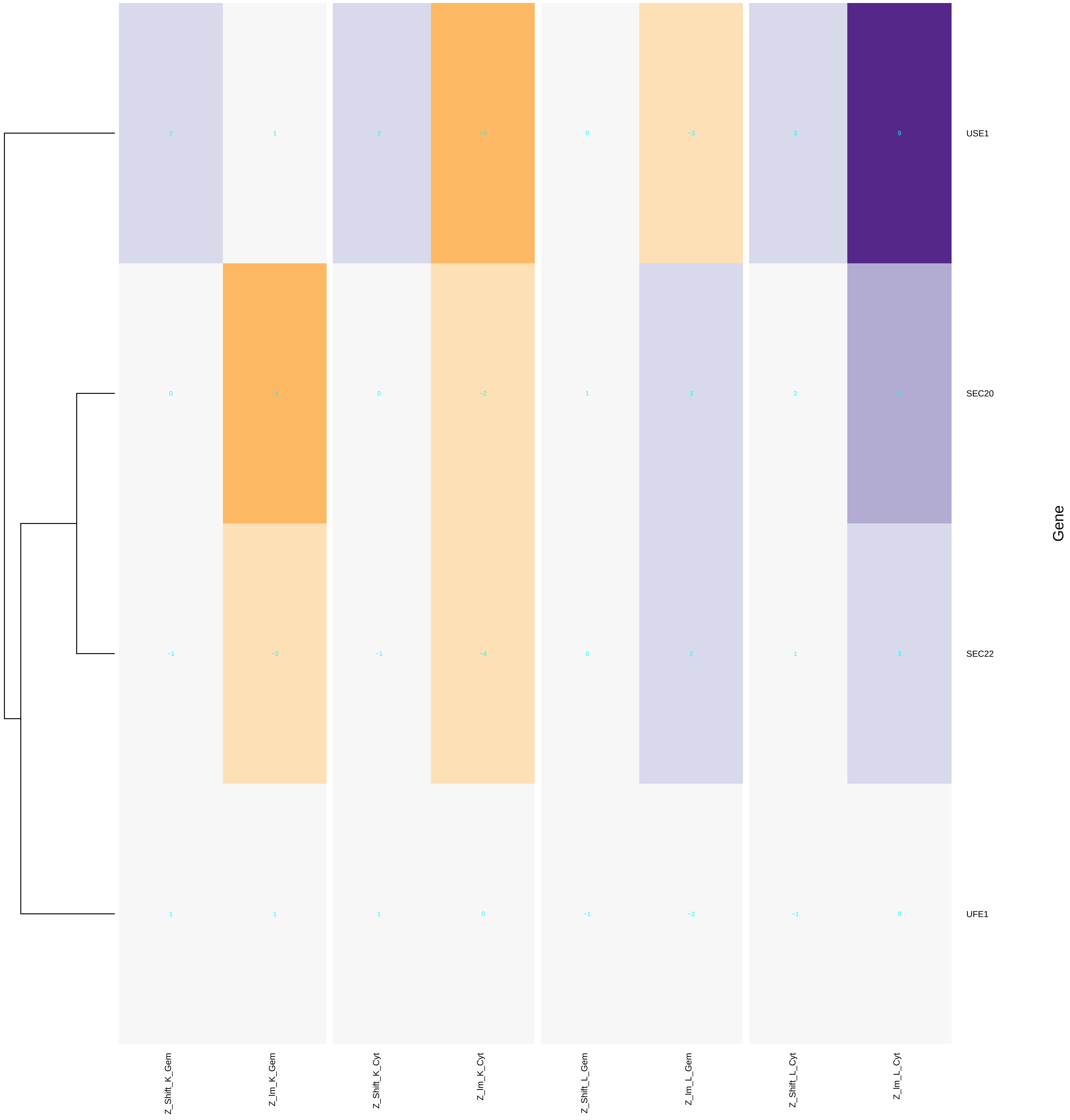

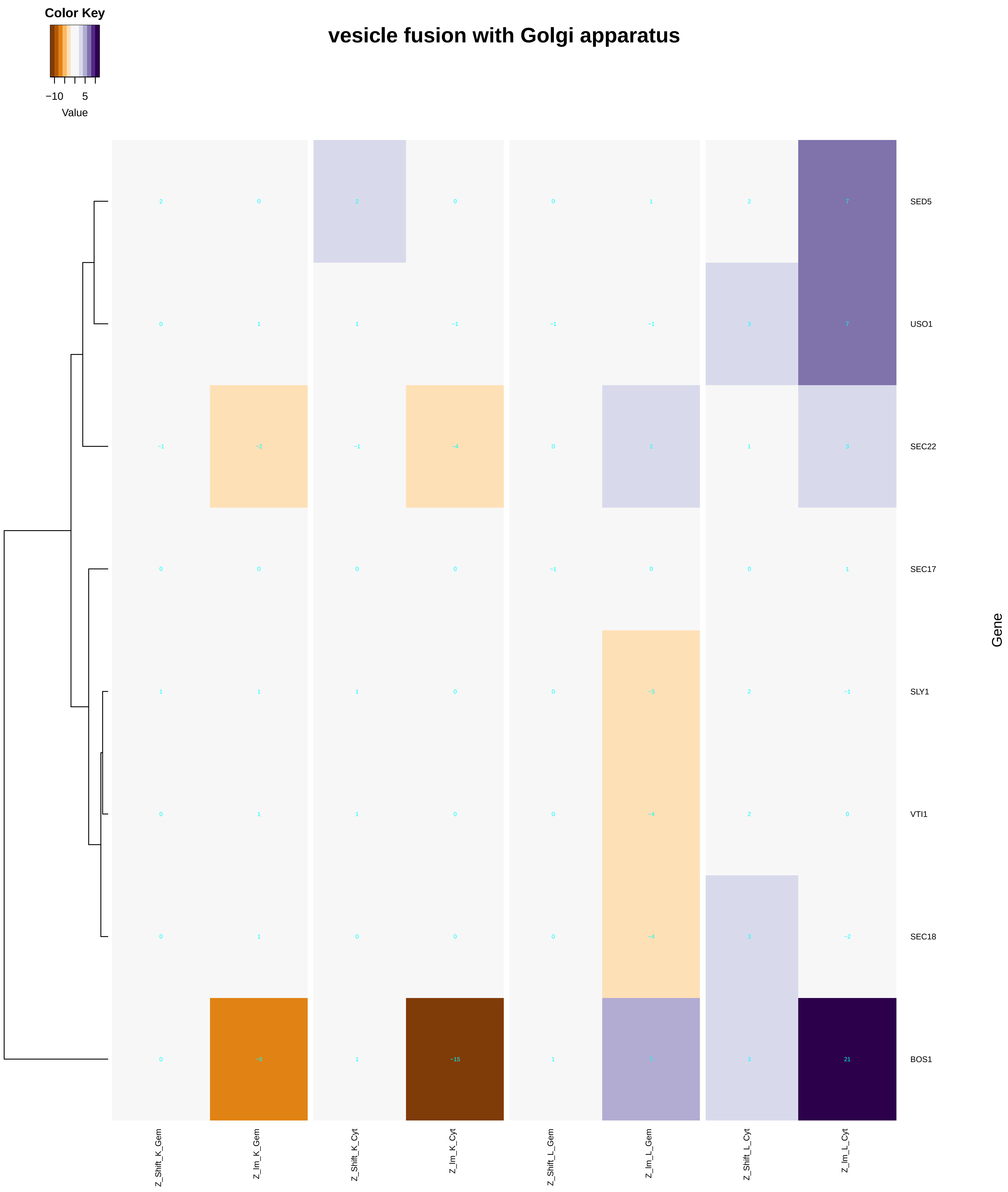
vesicle fusion with Golgi apparatus
Color Key
−10
5
Value
SED5
USO1
SEC22
SEC17
SLY1
VTI1
SEC18
BOS1
Gene
Z_Shift_K_Gem
Z_Im_K_Gem
Z_Shift_K_Cyt
Z_Im_K_Cyt
Z_Shift_L_Gem
Z_Im_L_Gem
Z_Shift_L_Cyt
Z_Im_L_Cyt
